# Astroglial Networks and Implications for Therapeutic Neuromodulation of Epilepsy

**DOI:** 10.3389/fncom.2012.00061

**Published:** 2012-08-29

**Authors:** Mark R. Witcher, Thomas L. Ellis

**Affiliations:** ^1^Department of Neurosurgery, Wake Forest UniversityWinston-Salem, NC, USA

**Keywords:** astrocyte, tripartite synapse, neuromodulation, epilepsy, deep brain stimulation, vagal nerve stimulation

## Abstract

Epilepsy is a common chronic neurologic disorder affecting approximately 1% of the world population. More than one-third of all epilepsy patients have incompletely controlled seizures or debilitating medication side effects in spite of optimal medical management. Medically refractory epilepsy is associated with excess injury and mortality, psychosocial dysfunction, and significant cognitive impairment. Effective treatment options for these patients can be limited. The cellular mechanisms underlying seizure activity are incompletely understood, though we here describe multiple lines of evidence supporting the likely contribution of astroglia to epilepsy, with focus on individual astrocytes and their network functions. Of the emerging therapeutic modalities for epilepsy, one of the most intriguing is the field of neuromodulation. Neuromodulatory treatment, which consists of administering electrical pulses to neural tissue to modulate its activity leading to a beneficial effect, may be an option for these patients. Current modalities consist of vagal nerve stimulation, open and closed-loop stimulation, and transcranial magnetic stimulation. Due to their unique properties, we here present astrocytes as likely important targets for the developing field of neuromodulation in the treatment of epilepsy.

## Introduction

Epilepsy is a common chronic neurologic disorder affecting 0.5–1% of the population with an estimated lifetime risk greater than 4% (Hauser et al., 1993; Hesdorffer et al., 2011). More than one-third of all epilepsy patients have incompletely controlled seizures or debilitating medication side effects in spite of optimal medical management (Sander, 1993; Kwan and Brodie, 2000; Sillanpaa and Schmidt, 2006). Medically refractory epilepsy is associated with excess injury and mortality, psychosocial dysfunction, and significant cognitive impairment (Brodie and Dichter, 1996). Treatment options for these patients include new antiepileptic drugs (AEDs), which may lead to seizure freedom in a small percentage of patients (Fisher, 1993; Engel, 2012) and resective surgery which is associated with long term seizure freedom in 60–80% of patients (Engel et al., 2003; Lee et al., 2005b; Engel, 2012). The cellular mechanisms underlying seizure activity are incompletely understood, though multiple lines of evidence support the contribution of astrocytic cells, both individually and in networks. Many properties of astrocytes also make them important targets for the developing field of neuromodulation in the treatment of epilepsy.

## Astrocytes in Epilepsy

Historically, astroglia were thought to provide only metabolic and physical support for neurons. They serve as the primary source of energy for neurons (Brown et al., 2004) and serve to control ionic homeostasis and neuronal excitability by buffering potassium (Kofuji and Newman, 2004). It is now clear, however, that astroglia are directly involved in neuronal signaling, even locally at synapses (Barres, 1991; Bergles et al., 1997; Volterra et al., 2002; Hatton and Parpura, 2004; Lin and Bergles, 2004; Allen and Barres, 2005; Volterra and Meldolesi, 2005). Astroglia synthesize and recycle glutamate (Hertz and Zielke, 2004) and respond to synaptic release of neurotransmitters with both calcium waves and release of gliotransmitters that can further influence synaptic activity (Cornell-Bell et al., 1990a; Grosche et al., 1999; Schipke and Kettenmann, 2004; Pascual et al., 2005; Perea and Araque, 2005; Zorec et al., 2012), with important implications in the epileptic brain (Carmignoto and Haydon, 2012). Perisynaptic astroglial processes may detect spill out of glutamate and other substances from active synapses (Rusakov and Kullmann, 1998; Diamond, 2005), and respond structurally by extending and modifying their processes (Cornell-Bell et al., 1990b; Hirrlinger et al., 2004; Witcher et al., 2007, 2010). Variation in synapse strength and the degree to which substances escape the perimeter might determine whether astroglial processes grow toward and ensheath parts of some synapses and avoid or retract from others (Cornell-Bell et al., 1990b; Hatton and Parpura, 2004; Witcher et al., 2007). Astroglia also secrete substances that are critical to the formation and function of synapses during development (Mauch et al., 2001; Ullian et al., 2001, 2004; Christopherson et al., 2005; Goritz et al., 2005) and contain contact-mediated factors that influence synapse maturation (Mazzanti and Haydon, 2003; Murai et al., 2003; Hama et al., 2004).

Astrocytes also function as modulators of neurotransmitters. It is commonly accepted that astrocytes play a large role in glutamate uptake, mostly through the GLT-1 transporter (Danbolt, 2001), also known as the excitatory amino acid transporter-2 (EAAT2) transporter with assistance by additional uptake via the EAAT1 transporter (Shigeri et al., 2004). Astrocytes, through the regulation of extracellular glutamate diffusion, also appear to modulate intersynaptic crosstalk (Oliet et al., 2001, 2006; Theodosis et al., 2006) and modulate synaptic function (Piet et al., 2004). Astrocytic transporter placement may only be contributory to these effects, however, as glutamine synthetase plays a key role in the metabolism of uptaken glutamate (Magistretti, 2006). While glutamine synthetase has been shown to be upregulated in reactive astrocytes (Eddleston and Mucke, 1993), it appears to be reduced in the epileptic hippocampus (Eid et al., 2004; van der Hel et al., 2005), indicating potential changes in the uptake potential of astrocytic transporters. Glutamate transporters have similarly been shown to be reduced in the epileptic hippocampus (Proper et al., 2002). Similarly, GABA release from interneurons may be proportionately increased by intracellular astrocytic calcium through activation of the kainic subtype of neuronal ionotropic glutamate receptors (Liu et al., 2004). Disruption of this cycle could lead to a decrease in GABA release, and thus decreased inhibition of neurons. GABA and glutamate imbalance is believed to potentially be a key mechanism in pathologies such as epilepsy. Thus, astroglia likely modulate function of synapses individually and within synaptic networks.

## Astrocytic Change in Neuropathology Can Alter Function of Neuronal Networks

Multiple pathologically induced changes in astrocytic function have been described. For example, neuronal calcium homeostasis may be modulated by growth factors such as NGF, known to be released by astrocytes in response to excitotoxic injection, providing a means of protecting cultured neurons against excitotoxicity (Eddleston and Mucke, 1993). Extracellular levels of calcium, as well as the propagation of multicellular calcium waves may be tied intricately to mechanically induced stimulation in astrocytes (Ostrow and Sachs, 2005). Under pathological conditions, mechanical stimulation could be provided in the injured CNS by reactive gliosis, swelling, mass effects, or tissue hypertrophy (Ostrow and Sachs, 2005). Astrocytic dysregulation of the vascular system has also been implicated in the epileptic brain. For example, altered calcium signaling by astrocytes can result in changes in local vascular tone (Zonta et al., 2003; Gomez-Gonzalo et al., 2011). Additionally, the upregulation or induction of multiple inflammatory factors within perivascular astrocytes, including IL-1β, complement components, and plasminogen activator in the epileptic brain may also have dramatic effects on the vascular system leading to disruption of the blood brain barrier (as reviewed in Aronica et al., 2012). Dysfunctional astrocytic calcium signaling may also underlay the development of a recurrent excitatory loop sustaining ictal discharge (Gomez-Gonzalo et al., 2010). Astrocytic dysfunction can also result in neuronal oxidative stress (Takuma et al., 2004), as failed support of dopaminergic neurons has been detected after astrocytes were experimentally deprived of glutathione (Drukarch et al., 1997).

Pathological dysfunction has also been observed in the gap-junctional coupling of astrocytes. Along these lines, increases have been noted in the expression of connexin-43 in experimental excitotoxic injuries (Haupt et al., 2007) as well as from epileptic patient specimens (Naus et al., 1991). Upregulation may result in increased intercellular signaling through calcium waves (Scemes and Giaume, 2006), an increased potential for astrocytic glutamate release (Parpura et al., 2004), and may play a role in the potassium buffering capacity of astrocytes (Wallraff et al., 2006). Through gap junctions, astrocytes can exert neurotrophic and neuroprotective influences (Naus et al., 2001; Takuma et al., 2004). Conversely, gap junction communication can be reversed in pathological conditions such as hypoxia (Martinez and Saez, 2000), potentially resulting in increased neuronal injury (Ozog et al., 2002).

**Figure 1:**
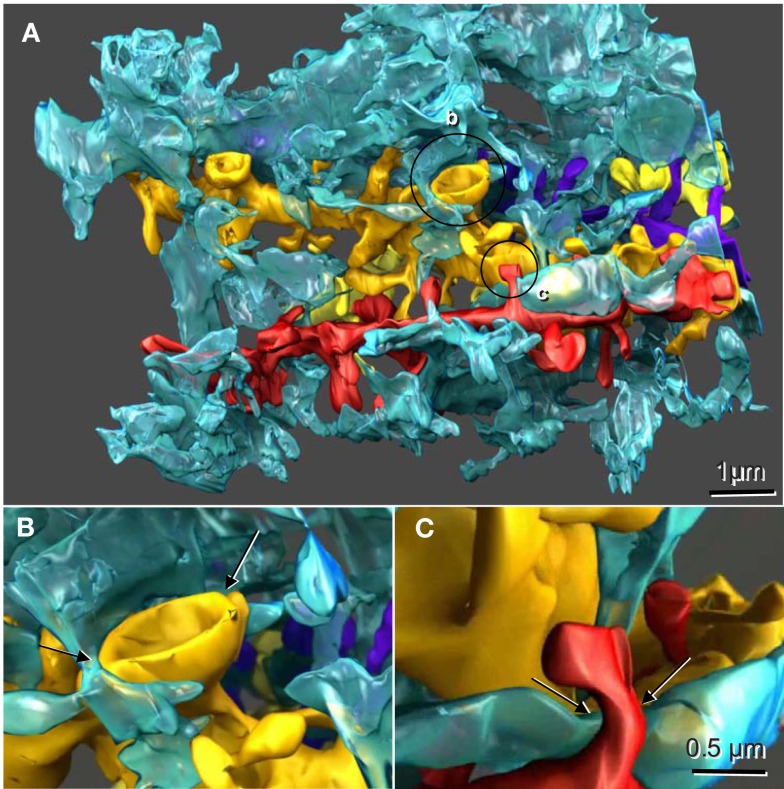
**Reconstructed dendrites, synapses, and associated astroglial processes from rodent hippocampus**. **(A)** Three-dimensional reconstruction of a single astroglial process (blue) interdigitating among many dendrites, four of which are reconstructed here (gold, yellow, red, and purple). Axonal boutons are not displayed. **(B)** Approximately 50% of the Axon-Spine Interface (ASI) of a mushroom spine was apposed by astroglia (arrows). **(C)** Only the neck of this thin dendritic spine was apposed by astroglia (arrows). Scale bar in **(C)** is for **(B)** and **(C)**. Figure reproduced with permission from John Wiley and Sons.

It is therefore clear that astroglial cells function to support the microenvironments of neuronal cells and to modulate neural networks. Given their unique structural interactions with multiple neuronal contacts simultaneously (Figure 1; originally published in Witcher et al., 2007; and Movie S1 in Supplementary Material), it is evident that astrocytes can readily integrate into a functional network. Astroglial cells may even function as separate, parallel networks in roles not currently understood. It is highly likely that astroglial networks have a fundamental role in disease states addressed through neurosurgical approaches. Specifically, astrocytes play a vital role in the epileptic brain, the treatment algorithm of which often ends in neurosurgical resection, transection, or disconnection of cellular networks. Similarly, multiple neurosurgical entities currently depend on the modulation of cerebral networks through introduction and manipulation of neuromodulatory devices such as deep brain stimulators. These techniques have analogous implications for astrocytes and the astroglial network. These disease processes include dystonia, movement disorders such Parkinson’s disease and psychological disorders dependent on appropriate modulation of network function such as Obsessive-Compulsive Disorder, depression, and others. Through a better understanding and manipulation of the astrocytic networks of the human brain, we hypothesize that new targets and modulatory therapies can be developed in the treatment of epilepsy.

## Implications in Epilepsy

One of the most consistently reported findings associated with seizure activity, whether a single event caused experimentally by focal application of convulsants (Jiang et al., 1998) or by repeated epileptic seizures *in vivo* (Scheibel and Scheibel, 1977), is hippocampal neuronal loss, subsequent deafferentation of dendritic partners, and loss of dendritic spines. Hippocampal alterations in mesial temporal lobe epilepsy (MTLE) include neuronal loss in the hippocampus, gliosis, and reorganization of subsets of neurons in the hippocampus (Sommer, 1880; Spencer, 2002). Lesions of CA1, in particular, are associated with the classically described Ammon’s Horn sclerosis (Mathern et al., 1997; Duvernoy, 2005), and greater loss of spines is associated with a greater degree of pathology. Other findings consistently reported in epileptic tissue include denuding of dendritic segments, as well as the formation of varicosities along dendritic shafts (Multani et al., 1994). The degree of synaptic loss can vary along individual dendrites (Scheibel and Scheibel, 1977). As reviewed by Fiala et al. (2002), these pathologies are also consistent with other neurodegenerative disorders, many of which cause deafferentation of dendrites. This observation led to the hypothesis that deafferentation may be the cause of dendritic spine pathology associated with disorders including epilepsy (Swann et al., 2000; Fiala et al., 2002). Structural evidence including the presence of giant spines, tortuous spines, an appearance of axon-free post synaptic densities (PSD) in dendritic spines, and aberrant synaptic partners (Raisman and Field, 1973; Baloyannis and Kim, 1979; Chen and Hillman, 1982) could support this hypothesis.

While recent attention has focused on changes in glial function in neurological diseases such as epilepsy (Tian et al., 2005; Binder and Steinhauser, 2006; Lee et al., 2006b; Matute et al., 2006), associated changes in perisynaptic astrocytic structure have been less of a focus, though changes in the amount of synaptic astrocytic contact would also be expected in the epileptic hippocampus. Important findings established or implicated in this form of epilepsy include increases in extracellular glutamate (During and Spencer, 1993), decreases in glutamate metabolism (Malthankar-Phatak et al., 2006), decreases in glutamate-stimulated GABA release (During et al., 1995), increased GABA transporter expression (Lee et al., 2006b), and increased lactate levels (Cavus et al., 2005), all of which can be associated with astrocytic dysfunction. While multiple studies have addressed the morphological changes of reactive astrocytes (Krishnan et al., 1994; van Paesschen et al., 1997; Mitchell et al., 1999; Briellmann et al., 2002) ultrastructural alterations remain understudied. Of primary importance is evidence which contradictorily supports both increased and decreased association between synaptic partners and astrocytic processes.

Multiple lines of evidence would support the possibility of increased perisynaptic astroglial apposition in the epileptic brain. The first is that in the epileptic hippocampus, microdialysis studies indicate that extracellular glutamate levels rise prior to and during ictal onset, peaking at levels associated with potential neurotoxicity (During and Spencer, 1993). Previous work has shown that astrocytes are responsible for the removal of extracellular glutamate, and that astrocytic presence can be neuroprotective (Rosenberg and Aizenman, 1989). Increased presence of perisynaptic astrocytic processes could therefore potentially serve as a mechanism by which to lower glutamate concentrations to baseline levels. It has also been shown that astrocytic processes are attracted to the neurotransmitter glutamate (Cornell-Bell et al., 1990b). The source of extracellular glutamate is likely synaptic in nature. It is therefore plausible that astrocytic processes would be drawn to these remaining synapses.

Further supporting increased apposition are the findings that astroglial contact stabilizes larger synapses (Witcher et al., 2007) and increases the efficacy of synaptic transmission (Pfrieger and Barres, 1997). The effects of increasing the amount of apposition at individual synapses is currently unknown, but previous research suggests that the amount of surround is not correlated to synapse size (Ventura and Harris, 1999) and that any amount of synaptic contact may be significant (Witcher et al., 2007). As synaptic loss is a known consequence of epilepsy, astrocytic apposition could function to stabilize remaining synapses.

Finally, previously reported increases in individual neurotransmitter receptor could support increased apposition, since MTLE is associated with an increase in glutamate receptors in the epileptic hippocampus. Human hippocampal astrocytes, similar to neurons, contain a wide variety of glutamate receptors, including AMPA receptors (Seifert et al., 2004; Matute et al., 2006), which are increased in CA1 in human epileptic tissue (Brines et al., 1997). Given the established concomitant decrease in neuronal density in epileptic hippocampi, it is plausible that this glutamate receptor increase is in the astrocytic component of the neuropil.

In contrast to the preceding evidence, other findings support the likelihood that perisynaptic astrocytic apposition could instead be decreased in epilepsy. One line of evidence lies in the morphological changes which take place subsequent to neuronal deafferentation. Due to the decrease in hippocampal neuronal density and concomitant dendritic pathologies consistently reported in both experimental and human epilepsy (Scheibel and Scheibel, 1977; Belichenko and Dahlstrom, 1995; Drakew et al., 1996; Thompson et al., 1996), it has been hypothesized that epilepsy serves as a model of neuronal deafferentation (Swann et al., 2000; Fiala et al., 2002). In the hippocampus, Schaffer collateral axons provide excitatory input from CA3 pyramidal neurons to CA1 pyramidal neurons via apical dendrites in CA1 stratum radiatum. In MTLE, where neuronal loss is prominent in both the hippocampal subfields of CA3 and CA1, loss of CA3 pyramidal neurons results in deafferentation of CA1 pyramidal neurons. As reported from other brain regions, astrocytic withdrawal can result in increased signaling between postsynaptic and presynaptic partners (Oliet et al., 2001), as well as increased signaling between adjacent synapses (Piet et al., 2004). Similarly, astrocytic withdrawal has been shown to regulate synaptic connectivity in the arcuate nucleus (Fernandez-Galaz et al., 1997) and similar effects have been implicated in the hippocampal dentate hilus (Luquin et al., 1993; Klintsova et al., 1995). Therefore, decrease in astrocyte surround at the synapse could be a compensatory mechanism useful for increasing axonal input to deafferented dendrites.

Modifications in the pattern of glutamate transporter expression in the sclerotic human hippocampus provide further support for astrocytic withdrawal from synapses. As many as five types of glutamate transporters have been described as present, and are designated as EAAT1–5, as reviewed by Danbolt (2001). The major glutamate transporters associated with hippocampal astrocytes include EAAT1 (GLAST), EAAT2 (GLT), and EAAT3 (EAAC) while the other EAAT subtypes are typically associated only with neurons (Danbolt, 2001). In addition to the astrocytic distribution of EAAT2, a presynaptic neuronal distribution was also shown in a subpopulation of excitatory hippocampal terminals, including the CA1 (Chen et al., 2002, 2004). In the CA1 subfield, approximately 60% of astrocytic membranes contain EAAT2 transporter proteins compared to approximately 30% of neurons (Chen et al., 2004). EAAT2 localizes in the cellular membrane of astrocytes (Danbolt, 2001), while in neurons it appears to label in the membrane as well as the cytoplasm (Chen et al., 2002, 2004). EAAT3 transporters, by contrast, appear to have a cytoplasmic distribution in both astrocytes and neurons (Danbolt, 2001). The distribution of EAAT1, however, is strictly astrocytic and localized almost exclusively to cytoplasmic membranes (Danbolt, 2001).

The EAAT1 transporter concentration does not vary between the soma and processes of astrocytes, but varies relative to neighboring structures, where concentration is increased along membranes apposing neuropil, and decreased in membrane apposing cellular somata, pial surfaces, or capillary endothelium (Danbolt, 2001). The EAAT1 transporter is therefore a reliable marker of astrocytic membrane in the neuropil, and can be particularly useful in the distal processes of astrocyte where GFAP filaments do not extend (Bushong et al., 2002, 2004). While the EAAT2 transporter is associated with the majority of glutamate uptake from the hippocampus, and a causative relationship has been shown between its knockout and the development of lethal, spontaneous seizures (Tanaka et al., 1997), its expression in neurons makes it non-specific to astrocytic membranes.

In the sclerotic human hippocampus, expression of the EAAT2 transporters are significantly decreased in CA1, and EAAT1 levels show a similar trend (Proper et al., 2002). Paradoxically, a well-known consequence of sclerosis is the hypertrophy of astrocytes, demonstrated repeatedly through expansion of the GFAP protein unique to astrocytic soma and perisomatic processes. This protein, however, is absent in small, distal astrocytic processes, such as those that appose synaptic membranes (Bushong et al., 2002). As the EAAT1 and EAAT2 transporters are localized in all portions of the astrocytic membrane regardless of distance from the soma, they serve as indicators of the astrocytic processes distal to the appearance of GFAP. While the decrease in EAAT2 is significant in TLE (Proper et al., 2002), the trend toward a concomitant decrease in astrocyte-specific EAAT1 suggests that the decrease is likely contributed to by astrocytic changes. Decrease in these transporters suggests a decrease in non-GFAP containing perisynaptic astrocytic processes, thereby supporting a potential withdrawal of these processes.

Using three-dimensional unbiased brick analysis, multiple interesting findings were recently shown from the human epileptic hippocampus (Witcher et al., 2010). Specifically, it was shown that synaptic loss was indeed associated with the process of epileptogenesis, and that synaptic density decreased as gliosis increased. It was also shown that the morphology of remaining synapses was altered, and in the severe epileptic state, normal-appearing neuronal spines were replaced by abnormal giant spines which likely have unique physiological properties. Remaining synapses did not show increased numbers of vesicles, refuting the idea that high extracellular glutamate (During and Spencer, 1993; Cavus et al., 2005) is likely based on decreased uptake of the neurotransmitter. Synapses remaining in the epileptic hippocampus were not restricted from intersynaptic communication (Witcher et al., 2010). Despite these changes, however, it was clear that the apposition of perisynaptic astroglia supported larger synapses (Witcher et al., 2007, 2010). Therefore, while the astroglia and synapses show fundamental changes in the pathologic brain, it is clear that positive benefits arise from the relationship.

Neurosurgical management of epilepsy is useful in patients who are refractory to optimized medical treatment (Engel et al., 2003; Lee et al., 2005b). Current modalities are dominated by resection or disconnection of epileptic cellular networks. Descriptions of modern methods and their psychosocial implications occupy a vast literature and include lesionectomies, anterior temporal lobectomy, amygdalohippocampectomy, extratemporal resection, and corpus callosotomy (Feindel et al., 2009; Wilson and Engel, 2010). There is also a developing role for stereotactic radiosurgery (Quigg et al., 2012). In spite of improvements in surgical technique, approximately 4% of patients will suffer death or permanent neurologic disability (ILAE, 1997). Moreover, more than one-third of patients will not be candidates for surgical resection (Kwan and Brodie, 2000). For patients who are not candidates for resective surgery, there are limited options. Neuromodulatory treatment, which consists of administering electrical pulses to neural tissue to modulate its activity leading to a beneficial effect (Witcher and Ellis, 2011), can be effective for these patients.

## Neuromodulation and Astroglial Implications

The interest in neuromodulation for neurological disorders is driven by a desire to discover less invasive surgical treatments, as well as new treatments for patients whose medical conditions remain refractory to existing modalities (Witcher and Ellis, 2011). Interestingly, the unique characteristics of astrocytes make them interesting targets in the developing field of neuromodulation. Specifically, the use of these technologies requires modulation of large neural networks, and likely involve modulation of or through astrocytes or astroglial networks. These mechanisms likely involve reactive astrocytosis, network manipulation, and modulation of the release of gliotransmitters.

Astrocytic processes are ubiquitous between cells and around excitatory synapses throughout the CNS (Witcher et al., 2007, 2010). This proximity of astrocytic processes to synapses allows synaptic placement of glutamate transporters at sites of glutamate release and also the ability to limit or delimit interactions between neighboring synapses (Witcher et al., 2007, 2010). Astrocytes have also been shown to express metabotropic receptors for many neurotransmitters, including glutamate, GABA, norepinephrine, and acetylcholine (Tritsch and Bergles, 2007). Astrocytic responses to the activation of these receptors implies sensing of neuronal function and results in oscillations or repetitive spikes in Ca2+, which likely has influence over neuronal network function (Di Castro et al., 2011; Takata et al., 2011). Gliotransmitters, namely ATP, d-serine, and glutamate are release in response to neuronal and astrocytic stimulation which also could cause neuronal network effects (Santello and Volterra, 2009; Halassa and Haydon, 2010). Mechanisms eliciting neuronal responses have been studied in both culture and slice models and include stimulation of metabotropic receptors, photolysis of caged IP3 or infused caged Ca2+, and repetitive depolarization of the astrocyte membrane presynaptic (Araque et al., 1998; Parri et al., 2001; Fiacco and McCarthy, 2004; Fellin et al., 2004; Jourdain et al., 2007).

Vagal nerve stimulation (VNS) is one example of neuromodulation that was developed in the 1980s, and which is now routinely available (Ben-Menachem, 2002). VNS, as an adjunct to medical management, may yield up to a 50% reduction in seizure frequency (VNSSG, 1995) although most of these patients will not be seizure free. Deep brain stimulation (DBS) is another example of neuromodulation. Given the significant experience and success of DBS for movement disorders (Krack et al., 2003) combined with its reversibility, programmability, and low risk of morbidity, there has been a resurgence of interest in using DBS devices for treating medically refractory epilepsy. Responsive neurostimulation (RNS) is a technology that detects seizure activity at a previously defined focus and applies an electrical stimulus to the site of seizure onset to terminate the seizure. Lastly, transcranial magnetic stimulation (TMS) is a nearly 25-year-old technology initially introduced as a means to non-invasively investigate corticospinal circuits. Currently, TMS is used primarily in clinical neurophysiology. Importantly, TMS can be used to evaluate and manipulate excitatory and inhibitory intracortical circuits with poststimulatory effect, allowing for a developing use in epileptic neuromodulation. A growing body of literature supports the involvement of astrocytes in the realization of therapeutic goals for each of these modalities, and will be reviewed below.

## Vagal Nerve Stimulation

The vagal nerve has a complex anatomical arrangement which projects to the autonomic and reticular structures and well as limbic and thalamic neurons. Stimulation of the vagus-nerve and its bilateral multisynaptic targets has become a common technology for the treatment of epilepsy. Over 50,000 patients have been treated with the technology, and current reports indicate an approximately 50% efficacy in seizure reduction, rivaling the efficacy of antiepileptic treatment, and often decreasing dependence on them (Labar, 2002). Efficacy has also been shown to increase over time (Vonck et al., 1999). The low side effect profile of VNS (Morris and Mueller, 1999) has also proven to be advantageous for users.

The mechanism of efficacy remains unknown, though certain structures within the brain appear to be affected by VNS. As evidenced by studies using positron-emission technology (PET), the thalamus is consistently affected by VNS stimulation, and blood flow to the cerebellum and cerebral structures is consistently altered (Ko et al., 1996; Henry et al., 1998, 1999; Ben-Menachem, 2002). Thalamic involvement has also been supported through SPECT (Van et al., 2000; Vonck et al., 2000) and functional MRI (Narayanan et al., 2002; Liu et al., 2003) analysis.

Studies of VNS have been reported from multiple vertebrate models including rodents (McLachlan, 1993), canines (Zabara, 1992), and lower primates (Lockard et al., 1990). In the rodent penicillin/pentylenetetrazol model, interictal spike frequency was reduced by 33% (McLachlan, 1993), the effect of which was later found to be greatest in continuous stimulation and reduced in a time-dependent fashion after stimulation (Takaya et al., 1996). Later tests showed that cortical excitability in rats can be modulated through VNS (De Herdt et al., 2010). Canine strychnine and pentylenetetrazol models show similar efficacy with lasting reduction in motor seizures and tremors (Zabara, 1992). In the alumina gel monkey model, seizures were eliminated in half of test animals during stimulation periods with some persistence into post-stimulation period (Lockard et al., 1990). Clinical trials have indicated seizure reduction at both low and high stimulation paradigms, with significantly greater reduction in the high stimulation group (Handforth et al., 1998) and overall efficacy showed a mean seizure reduction of approximately 35–45% (Morris and Mueller, 1999).

Astrocytic involvement in the regulation of the vagal nerve nuclei supports their importance in the efficacy of VNS. McDougal et al. (2011) recently demonstrated the activation of astrocytes within the nucleus of the solitary tract (NST) when afferent stimulation of the vagal nerve was applied. Using confocal, live-cell calcium imaging of brainstem slices, they showed that afferent activation of the vagal nerve resulted in increases in astrocytic intracellular calcium concentrations as well as in neurons. They then showed that the effect on astrocytes was blocked by the AMPA receptor antagonism and was unaffected by antagonism of NMDA and metabotropic glutamate receptors. This activation was dependent on extracellular Ca^2+^ influx through AMPA receptors. This Ca^2+^ influx was further amplified by calcium-induced calcium release via the ryanodine receptor. Selective staining verified the presence of the AMPAR subunit GluR1 on astrocytes. Taken together, they concluded that NST astrocytes may be active participants in the regulation of vagal activity (McDougal et al., 2011). This supports previous work which concluded that neurons in the NST are regulated via astrocytic glutamate signaling under pathologic and potentially physiologic conditions (Hermann et al., 2009).

## Direct Neural Stimulation

Neuromodulation through the direct implantation of chronic stimulating electrodes has become a standard of treatment in many neurological disorders. DBS lead implantation within the anterior nucleus of the thalamus (ANT), as well as other central nervous system (CNS) targets – including the caudate nucleus, centromedian nucleus of the thalamus, cerebellum, hippocampus, and subthalamic nucleus – results in seizure reduction in selected patients (Shandra and Godlevsky, 1990; Vercueil et al., 1998; Bragin et al., 2002; Lee et al., 2006a). In these studies, stimulation was delivered in an open-loop fashion, that is, in a pre-defined manner, independent of the momentary physiological activity of the brain. The exact mechanism of action of DBS in reducing seizure activity is, however, unknown. It is known that stereotactic lesions of the ANT in humans can result in reduced seizure frequency (Mullan et al., 1967). DBS may interfere with synchronized oscillations by neurotransmitter release (Lee et al., 2005a). Other evidence suggests that the most likely mechanism may involve stimulation-induced modulation of pathologic neural networks (McIntyre et al., 2004). High frequency DBS appears to reproduce the clinical effect of ablative procedures (Benabid et al., 1987). Moreover, at high frequencies, DBS may abolish cortical epileptiform activity (Lado et al., 2003). A microthalamotomy effect has been postulated based on the observation that some patients obtain reduction in seizure frequency prior to activation of the pulse generator (Andrade et al., 2006; Lim et al., 2007).

Although the precise mechanism by which DBS reduces seizure activity is unclear, inhibition of neurons immediately adjacent to the area of applied current is likely involved. A “reversible functional lesion” may be generated in structures integral to initiating or sustaining epileptic activity (Boon et al., 2007). The applied current may inhibit neurons with a pathologically lowered threshold of activation. Alternatively, DBS may act on neuronal network projections to nearby or remote CNS structures originating from the area of stimulation. This might take place through either activation of inhibitory projections or through the inhibition of excitatory projections.

As reviewed recently by Vedam-Mai et al. (2012), high frequency stimulation shows effect on astrocytic activity which has important implications in the role of astroglia in this modality. Astrocytes can be directly depolarized by stimulation (Kang et al., 1998) and have the potential to modulate local and distant neural networks using clinically relevant stimulation paradigms via the release of gliotransmitters including ATP and glutamate (Bekar et al., 2008; Tawfik et al., 2010).

Another important mechanism implicating astrocytes in clinical efficacy is reactive astrocytosis. Reactive astrocytosis is a well described phenomena of astrocytes at stimulator implant sites, and is defined as astroglial hypertrophy and upregulation of GFAP and other astrocytic proteins (Pekny and Nilsson, 2005). This finding was described initially in cats (Stock et al., 1979) and has since been described in multiple species including rats (Kraev et al., 2009), non-human primates (Griffith and Humphrey, 2006), and humans (Moss et al., 2004; Sun et al., 2008a; DiLorenzo et al., 2010; Vedam-Mai et al., 2011). In these series, reactive elements including multinucleated giant cells and macrophages were common findings. In human studies, common elements included thin glial rims surrounding the electrode tract; lymphocytes and monocytes have also been described near the electrode (Moss et al., 2004; DiLorenzo et al., 2010). The volume of the glial surround, which could greatly impact electrode function, is, however, not known (Moss et al., 2004). Investigation in rats, however, indicates regional variability in astroglial reactivity to implanted electrodes (Hirshler et al., 2010).

The effects on network activity by reactive astrocytosis then becomes an important focus of their overall effect. Reactive astrocytes display marked functional changes which could include direct neurotrophic effects though modified energetics or neurotrophic factor release, enhanced glutamate uptake, reorganization of metabolic pathways, and modulation of synaptic transmission (Liberto et al., 2004; Sofroniew, 2005; Escartin and Bonvento, 2008). Recently, a model using selective virus-induced reactive astrocytosis in rat hippocampal area CA1 demonstrated that astrocytosis resulted in specific deficits in inhibitory synaptic transmission, and caused disruptions in functional regulation of circuits resulting in enhanced excitability of the local network (Ortinski et al., 2010). Specifically, a reduction was found in elicited monosynaptic inhibitory responses, which led to a reduction in basal inhibitory neurotransmission without affecting intrinsic neuronal properties. This resulted from an alteration in the astrocytic glutamate/glutamine cycle which resulted in reduced synaptic GABA availability (Ortinski et al., 2010). Thus, a growing body of evidence supports that reactive astrocytosis at the electrode site could readily alter network effects of targeted neural circuits.

In contrast to open-loop stimulation, contingent or closed-loop stimulation is designed to suppress epileptiform activity by stimulating a defined epileptogenic target directly in response to detection of abnormal EEG activity. This form of closed-loop, responsive neural stimulation (RNS), has preliminarily been shown safe and efficacious (Sun et al., 2008b), and is currently being evaluated in a randomized trial to assess safety and efficacy in epileptic patients. While its experimental and clinical trial background are beyond the scope of this review, its’ similarities to DBS, notably contacting electrodes, pulse delivery, and network neuromodulatory effects imply a likelihood of astrocytic involvement analogous to open-loop stimulation.

## Transcranial Magnetic Stimulation

Transcranial magnetic stimulation of cortical tissues was initially reported by Barker et al. (1985) and quickly found acceptance as a research vehicle for neurophysiologists. TMS was initially applied to the study of the motor system (Barker et al., 1985) and has since expanded to include investigations in psychiatric conditions (Pascual-Leone et al., 1996), and migraine headache (Lipton and Pearlman, 2010). Importantly, it has also become a viable option for the treatment of drug resistant epilepsy. TMS exerts its effects through repetitive non-invasive stimulation in which a pulsed magnetic field creates current flow in the brain which can temporarily excite or inhibit target areas (Hallett, 2000).

The basis of TMS as a therapeutic neuromodulatory is derived from the lasting effects from the application of a train of transcranial stimuli. Theoretically, the lasting effects of TMS can be used to modulate activity in focal areas of cortex (Fregni and Pascual-Leone, 2007). The induced effect depends on the nature of the stimulation; that is, the frequency, the timing, the focus, and the intensity of the repetitive stimulation (Kimiskidis, 2010). While some paradigms have been studied using animal models, the numbers of basic studies particular to epilepsy are somewhat limited.

Early study within the mouse hippocampal-entorhinal cortex slice model indicated that repetitive direct (i.e., non-transcranial) stimulation at 1 Hz can depress the generation of ictal activity in a 4-aminopyridine model (Barbarosie and Avoli, 1997), in a frequency-dependent manner (D’Arcangelo et al., 2005). This frequency dependence has been replicated in TMS. Low-frequency TMS stimulation shows the tendency to lower seizure activity (Akamatsu et al., 2001; Godlevsky et al., 2006; Rotenberg et al., 2008). High frequency stimulation has been shown to potentially have both protective and inductive effects dependent on the chronicity of treatment and potentially other, unexplored, factors (Jennum and Klitgaard, 1996; Ebert and Ziemann, 1999).

Similar results have been identified in human studies. High frequency TMS has been shown to enhance cortical excitability at high intensities (Berardelli et al., 1998), while low-frequency TMS has been shown to reduce cortical excitability (Cincotta et al., 2003) as well as decreased strength of neuronal signaling (Muellbacher et al., 2000). As detailed by Kimiskidis (2010), the clinical effects are theoretically similar to long term potentiation (LTP) and long term depression (LTD) elicited by high- and low-frequency electrical stimulation, respectively. It is therefore possible that TMS at lower frequencies may exert its effect through the initiation of LTD, while at higher frequencies, the proconvulsant effect may be initiated through the induction of an LTP-type effect (Ziemann, 2005).

Direct evidence for astrocytic involvement in the neuromodulatory therapy is limited. Early work in a murine model found that high frequency TMS had a dramatic effect in the upregulation of astroglial gene expression (Fujiki and Steward, 1997) Following multiple high frequency trains (25 Hz), GFAP mRNA levels were significantly increased in the hippocampal dentate gyrus to levels similar to that following electroconvulsive seizures, indicating induction of an astrocytic reactive response (Fujiki and Steward, 1997). Indirectly, the analogous effects to LTD and LTP have important implications for astrocyte involvement, as the important contributions of astrocytes and gliotransmitters to synaptic plasticity have been described in multiple neuronal circuits (Yang et al., 2003; Witcher et al., 2007; Henneberger et al., 2010; Ben Menachem-Zidon et al., 2011; Bonansco et al., 2011; Navarrete et al., 2012).

## Conclusions and Future Directions

In spite of optimal medical management, many patients with epilepsy remain medically refractory and suffer from debilitating seizures. Some of these patients may benefit from neuromodulatory treatment. As the evidence above indicates, it is very likely that modulation of astroglial function is important to the efficacy of neuromodulation. Additional studies are needed to identify the appropriate patient populations for neuromodulation, optimal targets, optimal stimulation modalities, and paradigms. It is also critical that the cellular and network mechanisms underlying the effects of these treatments must be better elucidated. Further studies are needed to determine the contribution of neural and glial components of the nervous system, and future modalities must be developed which optimize both. Understanding these relationships may enable future technologies, perhaps even nanotechnologies, to flourish in the developing field of therapeutic neuromodulation.

## Conflict of Interest Statement

The authors declare that the research was conducted in the absence of any commercial or financial relationships that could be construed as a potential conflict of interest.

## Supplementary Material

The Supplementary Material for this article can be found online at http://www.frontiersin.org/Computational_Neuroscience/10.3389/fncom.2012.00061/abstract

Supplementary Movie S1 **Reconstructed dendrites, synapses, and associated astroglial processes from rodent hippocampus**. Note the spatial relationship of a single astrocytic process (blue) interdigitating among multiple unique dendrites (gold, yellow, red, and purple). Axonal boutons are not displayed. These spatial relationships likely allow for the interactions of astrocytes with neurons, the regulation of neuronal communication, and form the basis of the astroglial-neuronal network. Figure acknowledgment to Cosmocyte, Inc. (Savage, MD, USA) for production in rendering and display.
